# Microstructure, Characterization and Mechanical Properties of Coal and Coal-like Materials

**DOI:** 10.3390/ma16051913

**Published:** 2023-02-25

**Authors:** Xuesheng Liu, Yunliang Tan, Yunhao Wu, Xuebin Li

**Affiliations:** 1 College of Energy and Mining Engineering, Shandong University of Science and Technology, Qingdao 266590, China; 2 State Key Laboratory of Mining Disaster Prevention and Control Co-Founded by Shandong Province and the Ministry of Science and Technology, Qingdao 266590, China

Energy is the most basic driving force for world development and economic growth and the basis for human survival. Coal has long been a major component of global fuel supply, accounting for 27% of global energy consumption and 38% of the world’s total power generation [1]. Although the share of coal in global energy consumption is gradually decreasing, for most developing countries, the dominant position of coal in their energy field cannot be changed in the short term. As the main source of energy in industrial development and people’s daily lives, coal is widely used in various countries and regions [2,3].

With the gradual development of the coal industry, many scientists have found that different types of coal, such as coking coal, lignite and anthracite, have great differences in their mechanical properties and microstructure, and the mechanical response mechanisms and failure forms under different environments and conditions also have great differences [4,5]. Especially with the gradual increase of coal mining depth, coal is extremely vulnerable to damage and instability under the action of high temperature, high stress, high gas and mining disturbance, which endangers the safety and stability of underground production and the life safety of operators [6,7].

At present, the research related to coal structure mainly includes the macroscopic and microscopic characteristics of coal body and its classification, description method, pore structure, etc. [8]. The research of coal chemistry shows that the structure of coal determines the physical and chemical properties of coal, while the structure of coal rock mass controls the failure mechanism of coal rock mass; that is, the structural control of the catastrophic failure of coal rock in a general sense, and its failure form and strength are related to the shape of the structural plane and the physical properties of the materials.

Over the years, many scholars have conducted in-depth theoretical and experimental research on the structural behavior of coal rock failure. Based on the interaction between the shear strength of rock mass and the shear strength of the structural plane, Jaeger JC [9] explored the failure mode and strength index of a rock mass structure. Through the uniaxial compression test of the rock mass with penetrating joints, it was found that the rock mass with penetrating joints had slipped along the weak plane, causing vertical splitting and two forms of failure characteristics. The dip angle of the weak plane of the joint affected the failure form and overall strength of the structure. Nasseri [10] conducted a triaxial loading test on shale, and obtained the slip, shear and composite failure characteristics of a rock mass containing structural planes. Sun [11] believed that rock mass structure affects the mechanical properties of a rock mass and controls its deformation and failure. The control effect of a structure is much greater than that of rock material. This theory has been widely recognized by experts worldwide. Liu and Ma [12,13] found that the rock properties of the roof and floor slabs also have a great impact on the mechanical properties of coal, proposing a test method of coal mechanical properties considering the influence of roof lithology, and building a damage-constitutive model under different load conditions.

In addition, the high value-added utilization of tectonic coal resources can be effectively improved by studying the macroscopic and microscopic characteristics, classification and mechanical properties of coal and coal-like materials. In recent years, Paz-Ferreiro J [14] evaluated the multifractal characteristics of soil pores based on the mercury intrusion method and nitrogen adsorption method. Maria Mastal-erz and Sharon M Swanson [15,16] carried out a large number of pore determination experiments of coal samples. It was found that the pores in coal were mainly micropores and dominated the adsorption characteristics of coal samples. Larsen and Rahman [17,18] studied the adsorption process of organic compounds on the surface of a coal matrix and calculated the adsorption heat. Based on the mercury intrusion method and multifractal theory, Li analyzed the pore size distribution characteristics of tectonically deformed coals with different deformation degrees. The results showed that tectonic deformation leads to narrower pore size distribution, lower pore connectivity and more complex distribution of permeable pores in coal. These studies have explored the development direction of new coal sample materials, which is in line with the current trend of the global energy industry towards green, low-carbon and efficient utilization.

In order to further explore the structural characteristics, physical and chemical properties of coal and coal-like materials with the support of many experts and scholars worldwide, a Special Issue of *Materials* was launched, entitled ’Microstructure, Characterization and Mechanical Properties of Coal and Coal-Like Materials’. The aim is to show the latest scientific and technological achievements and cutting-edge testing techniques in the research of coal and coal-like materials, and to explore their microstructure characteristics, structural change rules and mechanical properties under various influencing factors. The Special Issue was launched on the 22 March, and 13 high-level papers have been included so far. Among them, five papers focus on the damage evolution and failure characteristics of coal and coal-like materials under different conditions. Four papers have studied the physical and chemical properties of new rock mass materials (coal gangue, coal tar pitch, freeze–thaw sandstone and anchored bedding rock mass). Three papers studied the crack propagation and seepage characteristics of coal rock mass, focusing on revealing its microstructure characteristics. In addition, some experts and scholars have used new experimental devices (CSS-1950 Biaxial Rheological Testing machine, the Split Hopkinson Pressure Bar system, Zeiss Xradia 510 Versa high-resolution CT system) to analyze the structural characteristics, physical and chemical properties of coal and coal-like materials, demonstrating great innovation. Promoting the development of cutting-edge science in the professional field, some experts and scholars have also studied the problems encountered in the field of coal mines and rock mass engineering based on the field engineering background, put forward a guiding field construction scheme and carried out industrial experiments, which have great engineering reference value.

The following are the main contents of this Special Issue:

## 1. Simulation Research on Impact Contact Behavior between Coal Gangue Particle and the Hydraulic Support: Contact Response Differences Induced by the Difference in Impacted Location and Impact Material (Yang Yang, Yao Zhang, Qingliang Zeng et al.) [19]

This paper studies the problem of coal gangue impacting hydraulic support in the process of top coal caving. The rigid–flexible coupling impact contact dynamic model between coal gangue and hydraulic support was established and the contact response difference in the support induced by the difference in impacted components and coal/gangue properties was compared and studied. The results show that the number of collisions, contact force, velocity and acceleration of impacted parts were different when the same single coal particle impacted different parts of the support. Various contact responses during gangue impact were more than 40% larger than that of coal, and the difference ratio could even reach 190%.

## 2. Deformation, Failure, and Acoustic Emission Characteristics under Different Lithological Confining Pressures (Shuo Wu, Guangpeng Qin and Jing Cao) [20]

In this paper, the influence of different surrounding rock pressure on the deformation and rock failure characteristics of a deep well drilling roadway is studied by uniaxial and triaxial compression testing of rocks. The results show that: the surrounding pressure has a significant effect on the damage deformation characteristics of the rock, and the change of the surrounding pressure directly affects the strength, damage form and elastic modulus of the rock; the strength limit of the rock increases with the surrounding pressure, and the damage form of the rock gradually changes to ductile damage with increase of the surrounding pressure; and the elastic modulus of the rock increases non-linearly with the increase of the surrounding pressure.

## 3. Inversion Method of the Young’s Modulus Field and Poisson’s Ratio Field for Rock and Its Test Application (Yanchun Yin, Guangyan Liu, Tongbin Zhao et al.) [21]

As a typical heterogeneous material, the heterogeneity of microscopic parameters of rock has an important influence on its macroscopic mechanical behavior. Based on digital image correlation (DIC) and the finite element method (FEM), a parameter field inversion method, namely the DF-PF inversion method, is proposed in this paper. The inversion accuracy was verified by numerical simulation and an indoor uniaxial compression test. The results show that compared with the traditional measurement method, the errors of macroscopic Young’s modulus and Poisson’s ratio calculated by the DF-PF inversion method were less than 2.8% and 9.07%, respectively. Based on the statistical analysis of Young’s modulus field and Poisson’s ratio field, the parameter uniformity and quantitative function relationship between microscopic parameters and principal strain can also be obtained in laboratory experiments. The DF-PF inversion method provides a new effective method for testing the Young’s modulus field and Poisson’s ratio field of rocks under complex stress conditions.

## 4. Damage Evolution Characteristics of Back-Filling Concrete in Gob-Side Entry Retaining Subjected to Cyclical Loading (Xicai Gao, Shuai Liu, Cheng Zhao et al.) [22]

Focusing on the influence of mining disturbances on the roadside support of gob-side entries being retained in deep coal mines, in this paper, uniaxial and cyclical tests of back-filling concrete samples were carried out under laboratory conditions to study damage evolution characteristics with respect to microscopic hydration. Understanding the characteristics of plastic strain, damage evolution and energy dissipation rate of filling samples has important reference value for real-time monitoring and failure warning of filling concrete in gob-side entry retaining.

## 5. Experimental Study of Energy Evolution at a Discontinuity in Rock under Cyclic Loading and Unloading (Wei Zheng, Linlin Gu, Zhen Wang et al.) [23]

To study the energy evolution of rock discontinuities under cyclic loading and unloading, cement mortar was used as a rock material and a CSS-1950 rock biaxial rheological testing machine was used to conduct graded cyclic loading and unloading tests on Barton’s standard profile line discontinuities with different joint roughness coefficients (JRCs). According to the deformation characteristics of the rock discontinuity sample, the change of internal energy was calculated and analyzed.

## 6. Comparative Study on the Seepage Characteristics of Gas-Containing Briquette and Raw Coal in Complete Stress–Strain Process (Ke Ding, Lianguo Wang, Zhaolin Li) [24]

Through triaxial compression and seepage experiments, the different damage forms of the two coal samples and the effect of their deformation and damage on their permeability were analyzed from the perspective of fine-scale damage mechanics. This is of great significance to explore the real law of coalbed methane migration.

## 7. Mechanical Properties and Failure Mechanism of Anchored Bedding Rock Material under Impact Loading (Yunhao Wu, Xuesheng Liu, Yunliang Tan et al.) [25]

In view of the problem that anchored bedding rock material is prone to instability and failure under impact loading in the process of deep coal mining, the instability criterion of compression and shear failure of anchored bedding rock material was established, and the instability criterion of compression and shear failure of anchored bedding rock material was obtained.

## 8. Multi-Level Support Technology and Application of Deep Roadway Surrounding Rock in the Suncun Coal Mine, China (Hengbin Chu, Guoqing Li, Zhijun Liu et al.) [26]

To solve these problems of poor supporting effect, serious deformation and failure of surrounding rock of roadways under deep mining stress, the authors analyzed the deformation and failure law of roadways surrounding rock under multi-stage support by numerical simulation and obtained the key parameters of multi-stage support. At the same time, industrial test verification was carried out on site. The research can provide reference and technical support for surrounding rock control of deep high-stress roadways.

## 9. The Influence of Coal Tar Pitches on Thermal Behaviour of a High-Volatile Bituminous Polish Coal (Valentina Zubkova and Andrzej Strojwas) [27]

In this paper, the influence of three coal tar pitches (CTPs), with softening points at 86, 94 and 103 °C, on the thermal behaviour of a defrosted high-volatile coal during co-carbonization and co-pyrolysis was studied. It was determined that CTP additives change the structure of the coal plastic layer, the thickness of its zones and the ordering degree of the structure of semi-cokes to a different extent and independently from their softening points.

## 10. Study on Influencing Factors of Ground Pressure Behavior in Roadway-Concentrated Areas under Super-Thick Nappe (Ruojun Zhu, Xizhan Yue, Xuesheng Liu et al.) [28]

In this paper, during the mining activity under the super-thick nappe formed by thrust fault, the law of mine pressure behavior was complex, and it was difficult to control the deformation and failure of the surrounding rock. Combined with the actual engineering conditions, the influence of different roof lithology conditions, the thickness of nappe, the mining height, the size of the barrier coal pillar and the creep time on mine pressure behavior was studied by UDEC numerical simulation software.

## 11. Influence of Microstructure on Dynamic Mechanical Behavior and Damage Evolution of Frozen–Thawed Sandstone Using Computed Tomography (Junce Xu, Hai Pu, Ziheng Sha) [29]

Frost-induced microstructure degradation of rocks is one of the main reasons for the changes in their dynamic mechanical behavior in cold environments. Computed tomography (CT) was performed to quantify the changes in the microstructure of yellow sandstone after freeze–thaw (F–T) action. In addition, the influence of the microscopic parameters on the dynamic mechanical behavior was studied. The research results can be a reference for constructing and maintaining rock structures in cold regions.

## 12. Mechanical and Microcrack Evolution Characteristics of Roof Rock of Coal Seam with Different Angle of Defects Based on Particle Flow Code (Qinghai Deng, Jiaqi Liu, Junchao Wang et al.) [30]

The angle of the defects has a significant influence on the mechanical characteristics and crack evolution of coal seam roof rock. In this paper, multi-scale numerical simulation software PFC2D was adapted to realize the crack propagation and coalescence process in the roof rock of a coal seam with different angles of defects under uniaxial compression. The study provides a certain reference for the use of various analysis methods in practical engineering to evaluate the safety and stability of rock samples with pre-existing defects.

## 13. A New Digital Analysis Technique for the Mechanical Aperture and Contact Area of Rock Fractures (Yong-Ki Lee, Chae-Soon Choi, Seungbeom Choi et al.) [31]

In this study, a new digital technique for the analysis of the mechanical aperture and contact area of rock fractures under various normal stresses is proposed. The proposed technique has the advantage of being able to analyze changes in the mechanical aperture and contact area under various normal stresses without multiple experiments. In addition, the change in the contact area on the fracture surface according to the normal stress can be analyzed in detail.
